# A Comparison of All-Cause Mortality in Patients Who Required Glaucoma Surgery for Neovascular Glaucoma or Primary Open-Angle Glaucoma: A Retrospective Cohort Study

**DOI:** 10.3390/vision9020049

**Published:** 2025-06-13

**Authors:** Laura D. Palmer, Levi D. Kauffman, Gregory B. Russell, Atalie C. Thompson, Gillian G. Treadwell

**Affiliations:** 1 Wake Forest University School of Medicine, Winston Salem, NC 27101, USA; ldkauffm@wakehealth.edu (L.D.K.); atathomp@wakehealth.edu (A.C.T.); gtreadwe@wakehealth.edu (G.G.T.); 2 Department of Surgical Ophthalmology, Atrium Health Wake Forest Baptist Medical Center, Winston Salem, NC 27157, USA; 3 Department of Biostatistics and Data Science, Wake Forest University, Winston Salem, NC 27101, USA; grussell@wakehealth.edu

**Keywords:** neovascular glaucoma, diabetic retinopathy, central retinal vein occlusion, primary open-angle glaucoma, mortality rate, glaucoma surgery, transscleral cyclophotocoagulation, tube shunt, cohort study

## Abstract

This retrospective review examines whether there is a difference in all-cause mortality in patients who required surgical intervention for neovascular glaucoma (NVG, N = 186) versus primary open-angle glaucoma (POAG, N = 190). Cox proportional hazard models compared mortality across three models: unadjusted, age-adjusted (Model 1), and age-, hypertension-, and diabetes-adjusted (Model 2). In all models, NVG patients who required glaucoma surgery had a higher all-cause mortality rate compared to those with POAG who underwent similar procedures: unadjusted (HR 2.22, (1.59, 3.10), *p* < 0.0001), Model 1 (HR 2.99, 95% CI (2.12, 4.22), *p* < 0.0001), and Model 2 (HR 1.88, 95% CI (1.27, 2.80), *p* < 0.0018). In Model 1, those with NVG due to PDR had a higher all-cause mortality rate after glaucoma surgery than those with NVG secondary to CRVO (HR 2.00, 95% CI (1.19, 3.45), *p* < 0.0095). Patients treated with CPC had higher all-cause mortality rates than those treated with tube shunt in all models: unadjusted (HR 1.82, 95% CI (1.33, 2.47), *p* < 0.0001), Model 1 (HR 1.91, 95% CI (1.40, 2.61), *p* < 0.0001), and Model 2 (HR 1.50, 95% CI (1.04, 2.16), *p* < 0.03). We observed a higher all-cause mortality rate among patients with NVG requiring glaucoma surgery compared to those with POAG requiring similar surgeries, which could suggest that NVG patients requiring glaucoma surgery had more compromised systemic health.

## 1. Introduction

Neovascular glaucoma (NVG) is a secondary glaucoma that accounts for 3.9% of all glaucoma diagnoses [1]. Chronic retinal ischemia precipitates NVG in 95% of cases [2]. Advanced cases of NVG are often refractory to treatment, with many patients developing severe visual impairment and, in some cases, a blind, painful eye [1]. Chronic retinal ischemia is most commonly secondary to diabetic retinopathy (DR) (33%), central retinal vein occlusion (CRVO) (33%), and ocular ischemic syndrome (OIS) (13%), but other causes include retinal artery occlusion, sickle cell retinopathy, or uveitic processes [2]. Many of these diseases are systemic, and therefore ocular ischemia may be accompanied by end-organ ischemia in other vital organs such as the brain, heart, or kidneys, thus providing insight into patients’ overall systemic health and risk for mortality.

Investigations regarding the relationship between glaucoma and mortality have demonstrated inconsistent results. A prior study utilizing the National Health Interview Survey found that glaucoma is associated with an increased risk of all-cause mortality and cardiovascular disease mortality [3]. Another study utilizing the National Health and Nutrition Examination Survey found no association between glaucoma and increased mortality [4]. However, neither of these studies assessed the association between types of glaucoma and mortality. Therefore, it is possible that different types of glaucoma portend varying mortality risks. A recent study found that patients with NVG had reduced survival compared to controls without glaucoma following tube shunt or cyclodestructive procedures [5]. However, this study did not directly compare survival of NVG to other forms of glaucoma. Insight on mortality can help guide clinical decision-making regarding how aggressively to treat the disease.

The goal of this study was to investigate all-cause mortality rates in patients with a diagnosis of neovascular glaucoma or primary open-angle glaucoma requiring surgical intervention (either tube shunt or transscleral cyclophotocoagulation (CPC)). Given that NVG is a manifestation of severe and poorly controlled microvascular disease, we hypothesized that patients with NVG requiring surgical intervention to treat glaucoma would have a higher all-cause mortality compared to patients with POAG who underwent similar surgical procedures. Additionally, this study sought to determine if the risk of all-cause mortality differed based on the specific vascular conditions that precipitated NVG or by the type of surgical procedures used to treat NVG.

## 2. Materials and Methods

This was a retrospective study using electronic medical record data from the Atrium/Wake Forest translational data warehouse. Institutional Review Board (IRB)/Ethics Committee approval was obtained, and this study adhered to the tenets of the Declaration of Helsinki. Informatics for Integrating Biology & the Bedside (i2b2) querying identified patients based on CPT and ICD codes. The i2b2 electronic database allows researchers to identify study cohorts from the Atrium/Wake Forest Baptist Hospital translational data warehouse. Researchers can extract data using the Data Puller Tool with IRB approval. All patients had neovascular glaucoma or primary open-angle glaucoma and underwent either tube shunt or transscleral cyclophotocoagulation (CPC) laser for management. The initial i2b2 query utilized CPT codes for procedures and ICD-9/10 codes for primary open-angle glaucoma or neovascular glaucoma (see Appendix A) and identified 384 patients with the aforementioned procedures and diagnoses codes. Inclusion criteria were defined as follows: patients who underwent CPC diode or tube shunt (Ahmed or Baerveldt) implantation between January 1, 2000 and January 1, 2019 and either a diagnosis of NVG (cases N = 186) or a diagnosis of POAG (controls N = 190). Patients with non-neovascular glaucoma, non-primary open-angle glaucoma, or an unclear diagnosis were excluded (N = 8), resulting in a total of 376 patients included for further review. The study parameters involved demographic factors such as age, gender, race, and ethnicity. Race data were combined into Black vs. non-Black race for the purpose of statistical analysis.

Additionally, date of surgery, surgical eye, type of surgery (Ahmed tube, Baerveldt tube, or CPC diode laser), etiology of NVG, date of death, and prior diagnosis of hypertension or diabetes mellitus were also documented when available. The date of surgery was utilized as the time point because patients requiring surgery had more severe glaucoma that could not be controlled medically.

Baseline group characteristics were summarized using means and standard deviations for continuous measures and frequencies and proportions for categorical variables. Independent Student t-tests were used to assess differences at baseline in continuous variables, while Fisher’s exact test was used for categorical group assessments. The primary outcome of survival after undergoing surgical intervention was compared between NVG and POAG. Length of survival was defined as date of surgery until death (an event) or last known date alive (censored as of this date). Survival status was determined based on Epic demographic data and published obituaries verifying name and date of birth. Kaplan–Meier survival estimates were calculated and plotted to demonstrate the estimated survival over the length of the study period; the log rank test of the chi-square approximation was used to assess survival differences between groups. Cox proportional hazards models were constructed to assess possible differences between study groups (NVG and POAG, NVG etiologies of CRVO and PDR, and surgical intervention methods) in three models: Model 0 was an unadjusted model, Model 1 adjusted for age alone, and Model 2 adjusted for potential confounders (age, hypertension (yes/no), and diabetes mellitus (yes/no)). Age, hypertension, and diabetes mellitus are associated with overall survival, and with a significant difference in age and rate of diabetes mellitus between POAG and NVG, the addition of these variables to the model adjusted for their effect on overall survival. Cox proportional hazards models were verified utilizing Schoenfeld residuals and the supremum test (the recommended approach to ASSESS with proportional hazard option in the SAS software) [6]. Graphics for direct adjusted survival estimates were created. Statistical significance was defined as *p* < 0.05. All statistical analyses were performed using SAS (version 9.4, Cary, NC, USA).

## 3. Results

A total of 384 patients who underwent glaucoma surgery and had a diagnosis of either NVG or POAG were initially identified. Eight patients were excluded due to unclear diagnosis or because they had other forms of glaucoma (i.e., not NVG or POAG). A total of 376 patients were eligible for review.

Patients with POAG ranged in age between 17 and 94 years, and patients with NVG ranged in age from 24 to 86 years. Patients with POAG were older than patients with NVG at the time of surgery (*p* < 0.001) (Table 1). There were no significant differences in sex, race, or ethnicity between POAG and NVG patients (Table 1).

The overall median survival was 9.8 years, with 161 deaths in 376 total patients, and with 2564 years of follow-up available for analysis. In addition, female vs. male sex (HR 0.91, 95% CI (0.66, 1.24) *p* = 0.54), Black vs. non-Black race (HR 0.95, 95% CI (0.69, 1.30), *p* = 0.73), and Hispanic vs. non-Hispanic ethnicity (HR 0.66, 95% CI (0.16, 2.70) *p* = 0.57) were not associated with survival.

### 3.1. NVG Versus POAG

**Figure 1:**
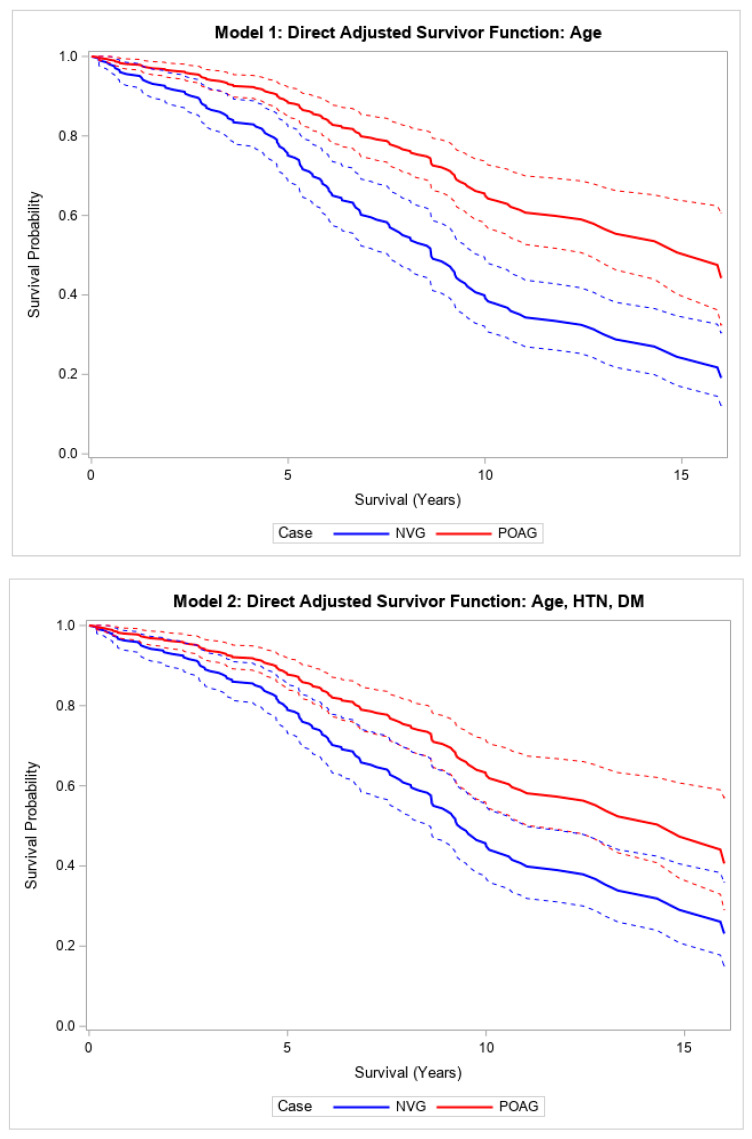
Adjusted models comparing survival after glaucoma surgery in patients with NVG versus those with POAG. Neovascular glaucoma (NVG); primary open-angle glaucoma (POAG); hypertension (HTN); diabetes mellitus (DM).

In the unadjusted model, patients with NVG requiring glaucoma surgery had higher all-cause mortality, or shorter survival, after surgery compared to patients with POAG requiring similar glaucoma surgeries (HR 2.22, 95% CI, (1.59, 3.10), *p* < 0.001) (Table 2; Supplemental Figure S1). This relationship was strengthened and remained significant after adjusting for age (HR 2.99, 95% CI (2.12, 4.22), *p* < 0.001) (Table 2; Figure 1). This relationship also remained statistically significant after fully adjusting for other covariates (HR 1.88, 95% CI (1.27, 2.80), *p* < 0.002) (Table 2; Figure 1). The median survival times were 7.8 and 11 years in patients with NVG and POAG, respectively, who underwent glaucoma surgery (Supplemental Table S1). A 5-year increase in age was associated with a 1.27 (95% CI (1.19, 1.36)) increase in the hazard of death in this age-adjusted model (*p* < 0.001).

Direct adjusted survival curves demonstrated increased all-cause mortality in patients with NVG compared to those with POAG after glaucoma surgery in the model adjusted for age (top) and the model adjusted for age, hypertension, and diabetes mellitus (bottom). The solid line provides the estimated direct adjusted survival estimate for each group, with the corresponding dashed colored lines representing the 95% confidence bounds.

### 3.2. NVG Secondary to PDR Versus CRVO

**Figure 2:**
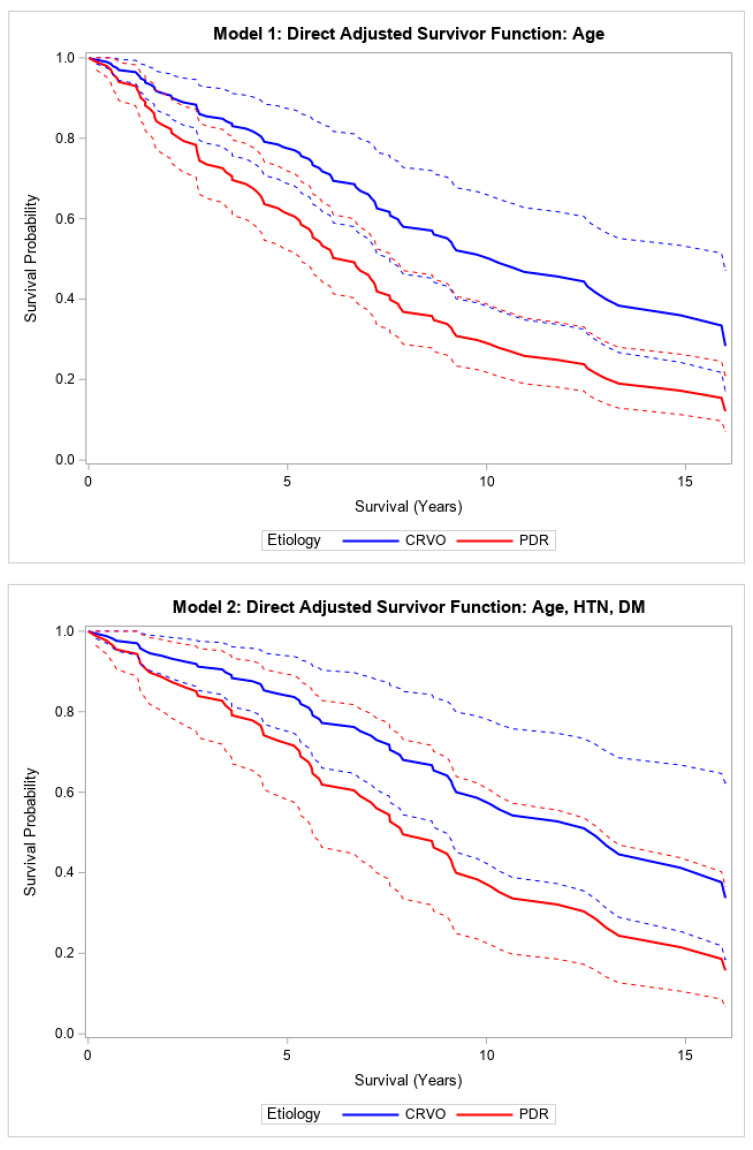
Adjusted models comparing survival after glaucoma surgery in patients with NVG secondary to PDR versus those with CRVO.

In this study cohort, the two most common causes of NVG were PDR (59.1%) followed by CRVO (23.1%) (Supplemental Table S2). PDR patients had an age range between 24 and 82 years, while CRVO patients had an age range between 45 and 86 years. When comparing PDR and CRVO as etiologies for NVG, the unadjusted model showed no significant difference in survival after glaucoma surgery. The HR of PDR vs. CRVO in the unadjusted model was 1.18 (95% CI (0.74, 1.96), *p* = 0.47). Patients with NVG secondary to CRVO (N = 44) had a mean baseline age of 67.5 years (SD 11), and patients with NVG secondary to PDR (N = 110) had a mean baseline age of 55 years (SD 13.6) with *p* < 0.001, indicating that adjustment for age was appropriate (Supplemental Table S3). After adjusting for age, there was a significant difference when comparing the survival of PDR patients to CRVO patients (*p* < 0.001). Adding age to the Cox regression model, the hazard of death comparing PDR to CRVO was 2.00 (1.19, 3.45) (*p* = 0.01) (Table 3, Figure 2). This relationship was not statistically significant after adjusting for age, hypertension, and diabetes mellitus (HR 1.94, 95% CI (0.76, 4.93), *p* = 0.16) (Table 3; Figure 2). Patients with NVG due to PDR had a median survival of 7.6 years compared to 10.9 years in those with NVG due to CRVO.

Direct adjusted survival curves demonstrated increased all-cause mortality in patients with NVG secondary to PDR compared to those with NVG secondary to CRVO after glaucoma surgery in the model adjusted for age (top) and the model adjusted for age, hypertension, and diabetes mellitus (bottom). The solid line provides the estimated direct adjusted survival estimate for each group, with the corresponding dashed colored lines representing the 95% confidence bounds. Central retinal vein occlusion (CRVO); proliferative diabetic retinopathy (PDR); hypertension (HTN); diabetes mellitus (DM).

### 3.3. CPC Versus Tube Shunt

**Figure 3:**
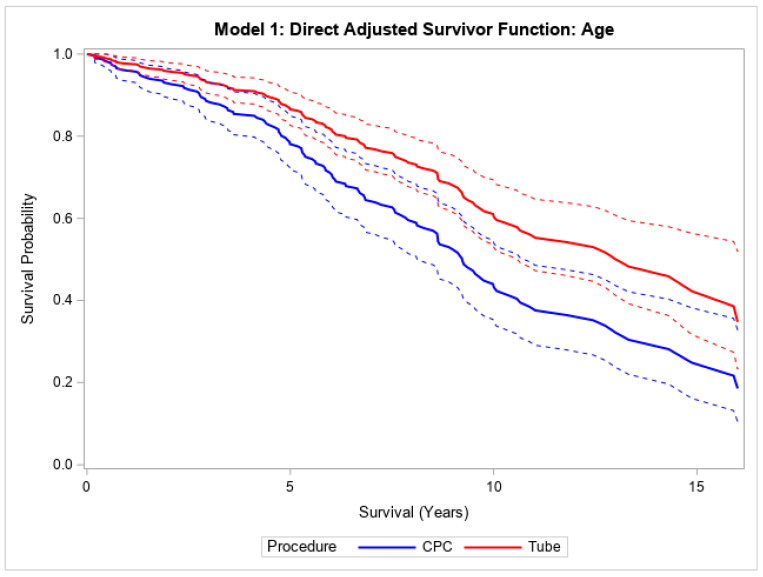

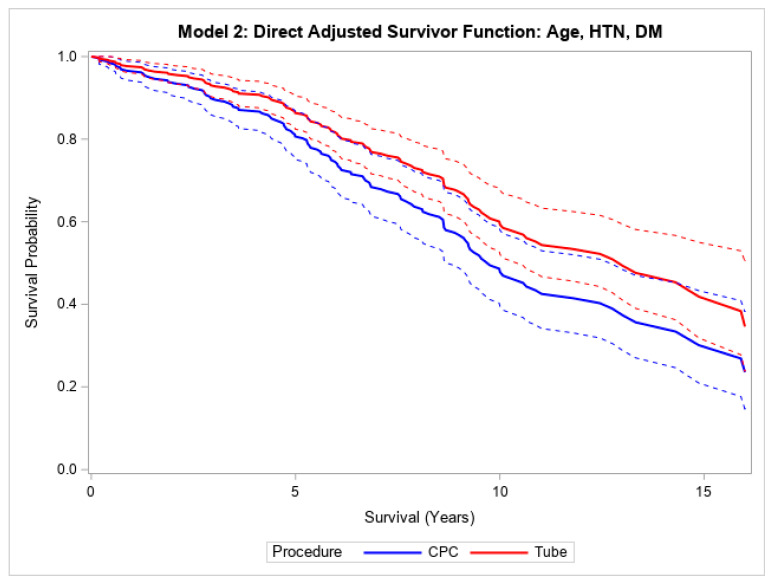
Adjusted survival curves comparing CPC diode laser versus tube shunt surgery.

Finally, in the overall cohort, patients with CPC had a higher mortality compared to those undergoing tube surgery, both without (HR 1.82, 95% CI (1.33, 2.47), *p* < 0.001) and with (HR 1.91, 95% CI (1.40, 2.61), *p* < 0.001) adjustment for age (Table 3; Figure 3). This relationship was still statistically significant after adjusting for age, hypertension, and diabetes mellitus (HR 1.50, 95% CI (1.04, 2.16), *p* = 0.030) (Table 3; Figure 3). Patients who underwent CPC laser treatment had a median survival of 8.3 years, and those who underwent tube shunt treatment had a median survival of 12.5 years. Within NVG patients, the median survival times were 7.6 and 8.5 years for CPC laser and tube shunt, respectively. In the POAG group, patients who received CPC laser treatment had a median survival of 9.5 years; 53.7% of the POAG patients who received tube shunt surgery were still alive at 10.9 years.

Stratifying by etiology (NVG or POAG) showed similar trends within each subtype, but the results were too underpowered to reach statistical significance. A much higher proportion of patients with NVG than those with POAG underwent CPC.

Direct adjusted survival curves demonstrated increased mortality in patients who underwent CPC diode laser surgery compared to patients who underwent tube shunt surgery in the model adjusted for age (top) and the model adjusted for age, hypertension, and diabetes mellitus (bottom). The solid line provides the estimated direct adjusted survival estimate for each group, with the corresponding dashed colored lines representing the 95% confidence bounds. Cyclophotocoagulation diode laser (CPC); hypertension (HTN); diabetes mellitus (DM).

## 4. Discussion

This study demonstrated that patients with NVG who underwent glaucoma surgery (tube shunt or CPC laser) had a higher all-cause mortality rate in the period after surgery relative to patients with POAG who underwent similar surgical procedures. Additionally, the two most common etiologies for NVG in this study cohort were PDR and CRVO, which reaffirms prior research illustrating the central role of chronic retinal ischemia in progression to NVG [1]. Furthermore, patients with NVG secondary to PDR had a lower probability of survival in the period after glaucoma surgery than patients with NVG secondary to CRVO. In patients who develop NVG, especially those who develop it due to PDR, more aggressive disease management and tighter blood glucose control may be warranted to reduce mortality risk. Finally, in the overall cohort, those treated with CPC laser had a higher all-cause mortality rate than those who received tube shunt surgery. This may be related to the use of CPC laser in patients with more advanced or refractory disease, who may have more significant comorbidities.

A previous study by Zhou et al. compared five-year survival rates after surgical intervention (tube shunt or cyclodestructive procedures) in patients with NVG within the Minnesota Veterans Affairs Healthcare System to age- and sex-matched controls from the general Minnesota population [5]. This study found that patients with NVG had a lower five-year survival rate after surgical intervention compared to the controls (62% versus 80%). Our study expands upon these findings by illustrating that different types of glaucoma requiring surgery are associated with variable all-cause mortality risks. In this study, patients with NVG had a lower rate of survival in the time after surgery compared to their POAG counterparts who underwent similar procedures in both the unadjusted and age-adjusted models. In the unadjusted model, those with NVG were twice as likely to die after surgery compared to those with POAG. When adjusting for age, patients with NVG had a three-times-higher all-cause mortality rate in the period following surgery compared to those with POAG. After adjusting for age, hypertension, and diabetes mellitus, we still observed a significant disparity in survival.

It is possible that disparities in overall systemic health could explain the differing mortality rates we observed here. Prior research indicates that patients with NVG are more likely to have advanced stages of other systemic diseases. For example, a prior study identified several systemic diseases that are associated with an increased risk for developing a CRVO, which is a well-studied risk factor for developing NVG [7]. Specifically, those with end-organ damage due to diabetes had a significantly higher risk of developing a CRVO compared to patients with DM without end-organ damage [7]. This same study found that patients with end-organ dysfunction secondary to hypertension had a significantly higher risk for developing a CRVO compared to those with uncomplicated hypertension [7]. These findings emphasize that patients with systemic comorbidities are at a higher risk of developing a CRVO, which can subsequently lead to NVG. In addition, a few studies suggest that NVG may increase the risk for developing other systemic conditions that may affect mortality. For example, Su et al. found that NVG is associated with a higher risk for developing an ischemic stroke [8]. Our study observed a higher proportion of diabetics among those with NVG than among those with POAG. However, further studies are necessary to elucidate the relationship of NVG with developing other systemic diseases that could contribute to all-cause mortality.

Our results indicate that the risk of all-cause mortality among patients with NVG undergoing glaucoma surgery varied based on the underlying etiology. In this study’s cohort, patients who underwent glaucoma surgery for NVG secondary to PDR had a lower 10-year survival rate compared to those who underwent glaucoma surgery for NVG secondary to CRVO (Supplemental Table S3). Surprisingly, the 10-year survival rate for NVG is similar to the 10-year survival rate for some common cancers including colon, rectum, oral cavity and pharynx, leukemia, ovary, and lung and bronchus (Supplemental Figure S2) [9,10]. While NVG is not the direct cause of death in these patients, NVG is a marker of end-organ damage due to severe microvascular disease and so may reflect higher risk of death due to other complications of diabetes and hypertension [7]. The increased all-cause mortality observed in these patients is likely due to the uncontrolled underlying condition, such as diabetes, rather than the diagnosis of NVG or the surgery itself. Understanding the increased all-cause mortality rate associated with NVG may help guide clinical decision-making, and physicians may want to consider the life expectancy of patients with NVG when considering whether a patient should undergo surgery for glaucoma management.

Additionally, in exploratory sub-analyses, when comparing the two most common etiologies in this study, patients with NVG due to PDR were more likely to die in the period after glaucoma surgery relative to those with NVG secondary to CRVO after adjusting for age. Given that NVG may be an advanced presentation of PDR, these findings expand upon prior research illustrating the relationship between diabetic retinopathy (DR) and all-cause mortality. A review article performed by Zhu et al. found that the presence of DR, regardless of severity or staging, was associated with an increased risk for all-cause mortality compared to diabetic patients without DR [11]. Further investigations to understand the relationship between DR and mortality have focused primarily on the association between DR and cardiovascular disease and stroke risk. A prior study conducted by Klein et al. found that severe retinopathy or visual impairment can indicate the risk of death from ischemic heart disease in diabetic patients [12]. A more recent study expanded upon previous findings and highlighted that DR increases the risk for coronary heart disease, macrovascular events, and all-cause mortality in patients diagnosed with type 2 diabetes mellitus [13]. Other studies suggest that the presence of DR increases the risk of ischemic stroke in diabetic patients [14,15]. Another study conducted by Shukla et al. concluded that diabetic tractional retinal detachment requiring vitrectomy was a marker of poor long-term survival [16]. In this study, diabetic patients who received vitrectomy for tractional retinal detachments were found to have a 48.7% long-term, all-cause mortality rate over 10 years and a mean survival of 2.7 years [16]. These findings are similar to our study which indicated that patients with PDR undergoing surgical intervention for NVG have poor survival and are more likely to have diabetes. Finally, a recent study demonstrated that PDR predicted mortality [17]. This aligns with our findings because PDR was the most common cause of NVG in our study, and those with both NVG and PDR had a higher mortality following surgery than those with NVG and CRVO. Together, these findings could suggest that NVG requiring surgery, especially if NVG is due to PDR, may be a marker of end-organ vascular disease that portends poor survival.

The association between DR and mortality risk mirrors the increased mortality risk in other organs affected by the microvascular complications of diabetes. Aside from ocular complications, diabetes commonly leads to peripheral ulcers, especially of the foot, and subsequent peripheral neuropathy [18]. The presence of foot ulcers and peripheral neuropathy leads to an increased risk for death in diabetic patients [18,19,20]. Similarly, diabetic kidney disease is also a risk factor for mortality in diabetic patients [21,22]. Given the increased mortality risk associated with the systemic microvascular complications of diabetes, these patients should be followed more closely by both primary care physicians and specialists such as ophthalmologists or nephrologists. More aggressive disease management, including lower blood glucose levels or lower intraocular pressure, should be considered in these patients to reduce mortality risk and maximize long-term survival. Further studies assessing intraocular pressure trends or baseline visual acuity may reveal other predictors of mortality.

When considering whether to escalate glaucoma management, ophthalmologists should consider initiating advanced care planning and goals of care discussions, especially for patients with NVG and systemic comorbidities, as these patients may not always benefit from aggressive treatment and may have a higher all-cause mortality risk secondary to compromised systemic health. Palliative care evaluations are less common in surgical patients compared to patients on medicine services [23]. Additionally, these consults are often delayed until a surgical patient is within days of death [23]. In some cases, earlier palliative care engagement can help preserve the quality of life for both the patient and their caregivers regardless of whether the patient elects to undergo aggressive surgical management.

This study has several limitations. As a retrospective review, this was an observational study, and we were limited to the cohort size and data available in the charts. Though we included 19 years of data, the sample size was modest and may not be entirely representative of patients with NVG or POAG elsewhere. A larger proportion of patients with NVG than of those with POAG had missing data for Hispanic ethnicity, which reflects how electronic health records may under-report the ethnic diversity of the population. Furthermore, the sample size in sub-analysis Model 2 was slightly smaller due to missing data from some patients. Therefore, the confidence intervals are slightly larger and can be interpreted with caution. Finally, we did not adjust for ocular parameters, such as visual acuity or visual field testing, which could quantify the degree of visual impairment in patients. Significant visual impairment is strongly associated with decreased mobility, loss of independence, and increased fall risk and may also contribute to the varying mortality rates [24,25,26,27]. Given this is an observational retrospective study, there will be residual and unmeasured confounding between the groups. While the findings are underscoring an observation that individuals have greater all-cause mortality risk following glaucoma surgery for NVG than for POAG, we are not stating that NVG predicts mortality independent of its etiology. It is possible that complications from diabetes or hypertension, which contribute to NVG, could explain a large proportion of the burden of mortality we observed in the patients undergoing surgery for NVG, and that NVG is a marker of end-stage organ damage occurring in multiple systems.

## 5. Conclusions

Patients who underwent glaucoma surgery for NVG had a higher all-cause mortality rate compared to patients who underwent glaucoma surgery for POAG. Patients treated with CPC laser had a higher all-cause mortality rate than those treated with tube shunt procedures. Patients who underwent glaucoma surgery for NVG secondary to PDR had an increased risk of all-cause mortality compared to patients who underwent glaucoma surgery for NVG secondary to CRVO. These findings could suggest that the presence of severe NVG requiring surgery, especially NVG due to PDR, a type of end-organ dysfunction due to severe microvascular ischemia, could be related to compromised systemic health, which may contribute to poor overall prognosis and low survival. While the increased all-cause mortality rate is not a direct consequence of the surgery or the diagnosis of glaucoma, having severe NVG requiring surgery may be an important surrogate marker for poor systemic microvascular health and may be related to higher risk for mortality. Further studies should investigate how end-of-life prognosis and other markers of survival can be incorporated into goals-of-care discussions with patients who have severe NVG requiring surgery, especially if due to PDR.

## Figures and Tables

**Table 1:** Baseline demographic and clinical characteristics of patients.

Mean (SD) or N (%)	POAG N = 190	NVG N = 186	*p*-Value
Age (years)	66.0 (13.3)	60.3 (13.9)	<0.001 *
Female sex	85 (44.7)	79 (42.5)	0.68
Race			
Black/African American	81 (43.1)	66 (35.7)	0.24
Non-Black	107 (56.9)	113 (64.3)	
Missing	2	1	
Ethnicity			
Non-Hispanic/Latino	174 (96.7)	118 (92.9)	0.18
Hispanic/Latino	6 (3.3)	9 (7.1)	
Missing	10	59	
Medical Conditions			
Diagnosed HTN	153/186 (82.3)	122/139 (87.8)	0.21
Diagnosed DM	72/186 (38.7)	117/141 (83.0)	<0.001 *
Procedure			
BVT	143 (75.3)	60 (32.3)	<0.001 *
CPC	31 (16.3)	112 (60.2)	
Ahmed	16 (8.4)	14 (7.5)	

Neovascular glaucoma (NVG); primary open-angle glaucoma (POAG); hypertension (HTN); diabetes mellitus (DM); Baerveldt tube shunt (BVT); cyclophotocoagulation diode laser (CPC); standard deviation (SD). Statistical significance is defined as *p* < 0.05, and an asterisk (*) denotes a statistically significant *p*-value. Means and standard deviations were used for continuous variables and frequencies and proportions for categorical variables.

**Table 2:** Hazard ratios for mortality in the primary analysis across three models.

	Model 0	Model 1	Model 2
**NVG vs. POAG ^1^**			
Hazard ratio (95% CI)	2.22 (1.59, 3.10)	2.99 (2.12, 4.22)	1.88 (1.27, 2.80)
*p*-value	<0.001 *	<0.001 *	<0.002 *

Statistical significance is defined as *p* < 0.05, and an asterisk (*) denotes a statistically significant *p*-value. Model 0: unadjusted; Model 1: adjusted for age; Model 2: adjusted for age, hypertension, and diabetes mellitus. When adjusting for hypertension and diabetes mellitus in Model 2, N decreased, as these data were not available for some patients. This resulted in slightly larger confidence intervals. Neovascular glaucoma (NVG); primary open-angle glaucoma (POAG). ^1^ NVG vs. POAG: N for Models 0 and 1: NVG (N = 186), POAG (N = 190). N for Model 2: NVG (N = 139), POAG (N = 186).

**Table 3:** Hazard ratios for mortality in sub-analyses across three models.

	Model 0	Model 1	Model 2
**CPC vs. Tube ^2^**			
Hazard ratio (95% CI)	1.82 (1.33, 2.47)	1.91 (1.40, 2.61)	1.50 (1.04, 2.16)
*p*-value	<0.001 *	<0.001 *	<0.030 *
**Within NVG:**			
**CPC vs. Tube ^3^**			
Hazard ratio (95% CI)	1.39 (0.93, 2.07)	1.37 (0.92, 2.05)	1.23 (0.74, 2.06)
*p*-value	0.11	0.12	0.42
**Within POAG:**			
**CPC vs. Tube ^4^**			
Hazard ratio (95% CI)	1.42 (0.76, 2.66)	1.15 (0.61, 2.17)	1.20 (0.62, 2.31)
*p*-value	0.27	0.67	0.59
**Within NVG:**			
**PDR vs. CRVO ^5^**			
Hazard ratio (95% CI)	1.18 (0.74, 1.96)	2.00 (1.19, 3.45)	1.94 (0.76, 4.93)
*p*-value	0.47	0.01 *	0.16

Statistical significance is defined as *p* < 0.05, and an asterisk (*) denotes a statistically significant *p*-value. Model 0: unadjusted; Model 1: adjusted for age; Model 2: adjusted for age, hypertension, and diabetes mellitus. When adjusting for hypertension and diabetes mellitus in Model 2, N decreased, as these data were not available for some patients. This resulted in slightly larger confidence intervals. Neovascular glaucoma (NVG); primary open-angle glaucoma (POAG); cyclophotocoagulation diode laser (CPC); proliferative diabetic retinopathy (PDR); central retinal vein occlusion (CRVO). ^2^ CPC vs. Tube: N for Models 0 and 1: CPC (N = 149), Tube (N = 233). N for Model 2: CPC (N = 111), Tube (N = 214). ^3^ Within NVG: CPC vs. Tube: N for Models 0 and 1: CPC (N = 112), Tube (N = 74). N for Model 2: CPC (N = 81), Tube (N = 58). ^4^ Within POAG: CPC vs. Tube: N for Models 0 and 1: CPC (N = 31), Tube (N = 159). N for Model 2: CPC (N = 30), Tube (N = 156). ^5^ Within NVG: PDR vs. CRVO: N for Models 0 and 1: PDR (N = 110), CRVO (N = 44). N for Model 2: PDR (N = 88), CRVO (N = 32).

## Data Availability

The datasets generated and/or analyzed during this study are available from the corresponding author upon reasonable request due to patient privacy.

## Supplementary Materials

The following supporting information can be downloaded at: https://www.mdpi.com/article/10.3390/vision9020049/s1, Supplemental Figure S1: Proportion of patients with NVG or POAG surviving after glaucoma surgery in an unadjusted model; Supplemental Table S1: Survival after glaucoma surgery at 2, 5, and 10 years in patients with NVG or POAG; Supplemental Table S2: Etiology of NVG in this patient cohort; Supplemental Table S3: Survival after surgery at 2, 5, and 10 years in patients with NVG secondary to either PDR or CRVO; Supplemental Figure S2: Comparing 10-year survival rates for the most common cancers and NVG secondary to PDR and CRVO.

## Abbreviations

The following abbreviations are used in this manuscript:

BVT	Baerveldt tube shunt
CI	95% confidence interval
CPC	Cyclophotocoagulation diode laser
CRVO	Central retinal vein occlusion
DM	Diabetes mellitus
DR	Diabetic retinopathy
HTN	Hypertension
HR	Hazard ratio
IOP	Intraocular pressure
i2b2	Informatics for Integrating Biology & the Bedside
NVG	Neovascular glaucoma
OIS	Ocular ischemic syndrome
PDR	Proliferative diabetic retinopathy
POAG	Primary open-angle glaucoma
SD	Standard deviation

## Appendix A

Initial ICD codes used to identify patients with NVG included glaucoma associated with vascular disorders (ICD9 365.63), other specified glaucoma (ICD10 H40.89), and glaucoma secondary to other eye disorders (ICD10 H40.5). ICD codes used to identify patients with POAG included ICD-9 365.11 and ICD-10 H40.11. Initial CPT codes used to identify glaucoma surgeries included tube shunt (CPT 66180) and transscleral cyclophotocoagulation (CPT 66710).
