# Supplementary material for: A Comparison of All-Cause Mortality in Patients Who Required Glaucoma Surgery for Neovascular Glaucoma or Primary Open-Angle Glaucoma: A Retrospective Cohort Study
Source: Vision (Basel). 2025 Jun 13;9(2):49. doi: 10.3390/vision9020049 (PMC12197471; doi:10.3390/vision9020049)
Supplement: Supplementary file 1 [file vision-09-00049-s001.zip › vision-3446854-supplementary.pdf]

Supplemental Digital Tables & Figures

Supplemental Figure S1: Proportion of patients with NVG or POAG surviving after glaucoma surgery in an unadjusted model

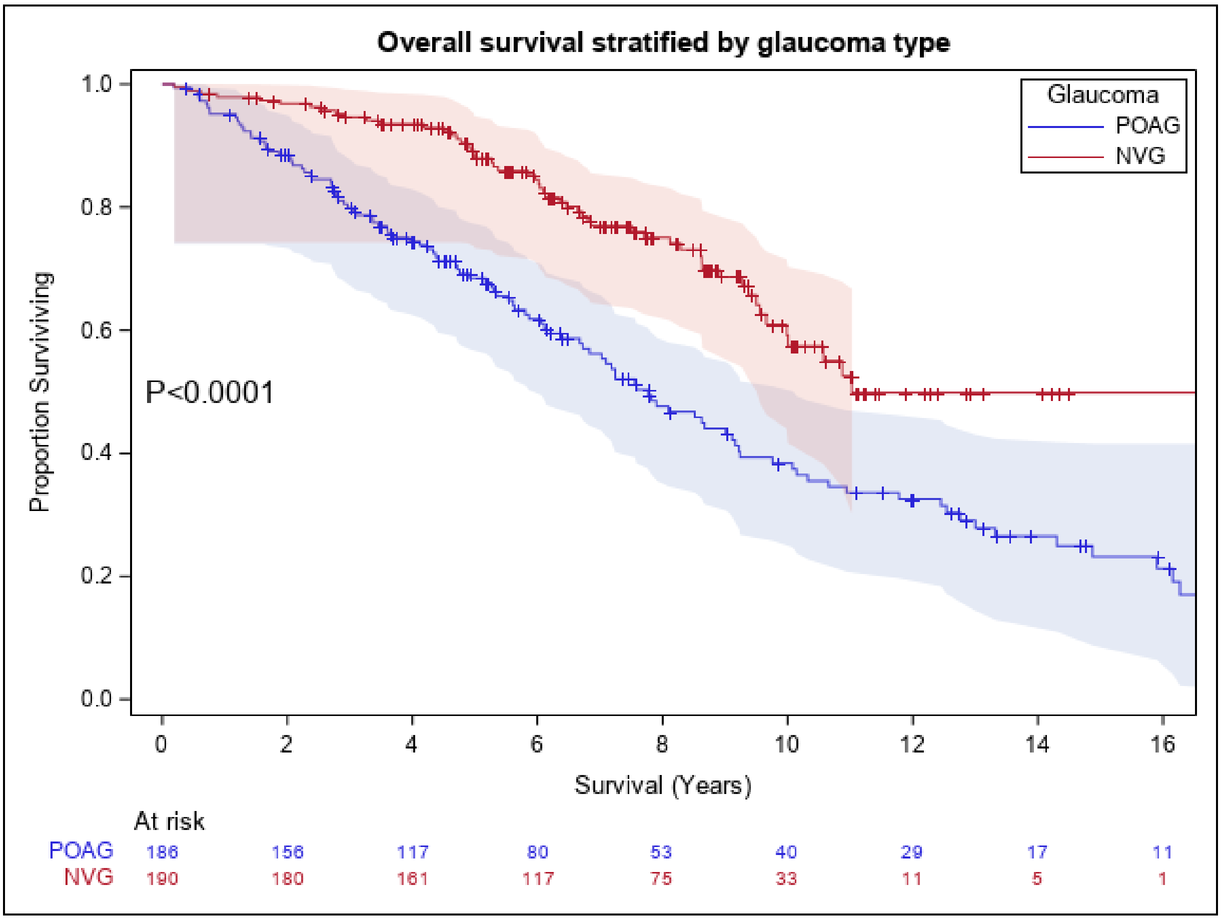

Neovascular glaucoma (NVG); primary open angle glaucoma (POAG)
Kaplan-Meier survival curve demonstrating decreased survival in patients with NVG than POAG after surgery without age adjustment.

Supplemental Table S1: Survival after surgery at 2, 5, and 10 years in patients with NVG or POAG

Group	Time from Surgery	Survival Estimate	Std. Error
**POAG (N=190)**	2 years	96.8%	1.3
	5 years	87.9%	2.5
	10 years	59.2%	5.1
	Median	11.0 years	
**NVG (N=186)**	2 years	88.5%	2.4
	5 years	68.5%	3.6
	10 years	38.4%	4.3
	Median	7.8 years	

Neovascular glaucoma (NVG); primary open angle glaucoma (POAG)

Supplemental Table S2: Etiology of NVG in this patient cohort

Etiology	NVG Cases (N=186)	Percentage
PDR	110	59.1%
CRVO	44	23.7%
CRAO	9	4.8%
OIS	3	1.6%
Other/Unspecified	20	10.8%

Neovascular glaucoma (NVG); proliferative diabetic retinopathy (PDR); central retinal vein occlusion (CRVO); central retinal artery occlusion (CRAO); ocular ischemic syndrome (OIS)

Supplemental Table S3: Survival after surgery at 2, 5, and 10 years in patients with NVG secondary to either PDR or CRVO

	CRVO (N=44)	PDR (N=110)	p-value
Mean patient age at time of surgery	67.5 SD (11.0)	55.2 SD (13.6)	p<0.001
2-year survival	81.6% (5.9)	91.6% (2.7)	p=0.81
5-year survival	71.6% (7.0)	73.0 % (4.5)	p=0.30
10-year survival	57.2% (8.5)	34.8% (5.6)	p=0.08
Median survival	10.9 years	7.6 years	

Central retinal vein occlusion (CRVO); proliferative diabetic retinopathy (PDR); standard deviation (SD)

Supplemental Figure S2: Comparing 10-year survival rates for the most common cancers and

NVG secondary to PDR and CRVO

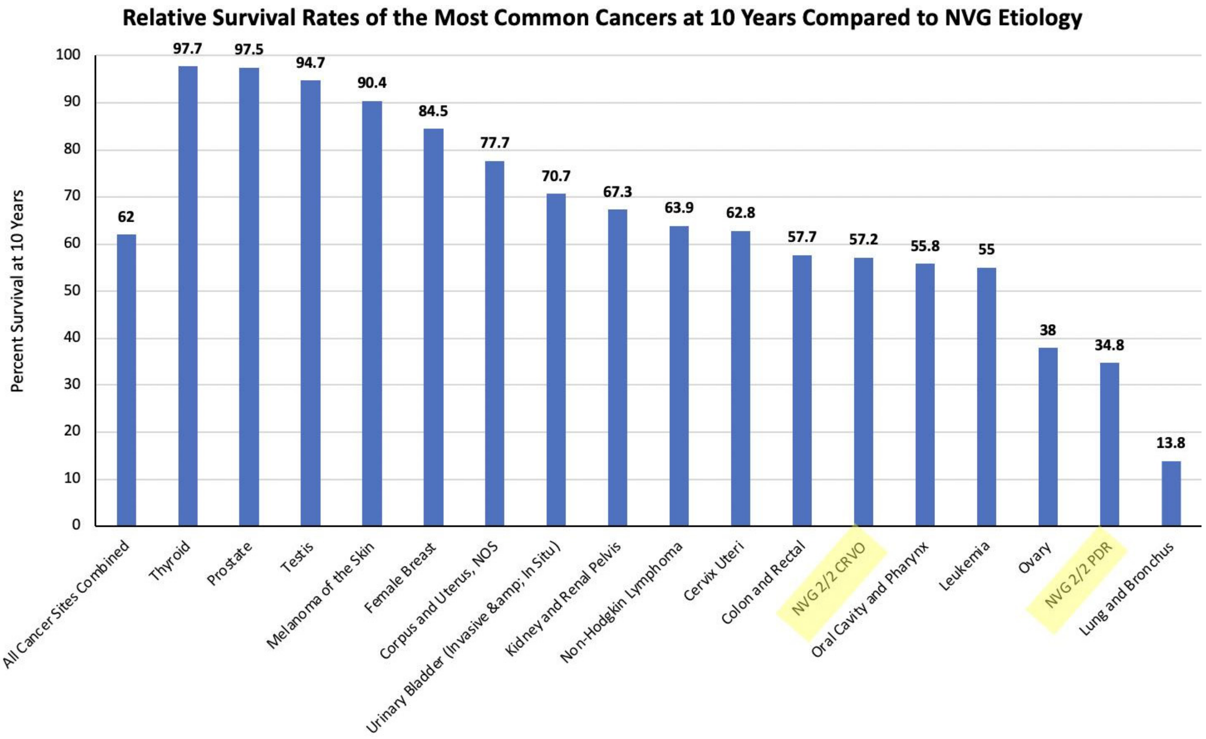

Neovascular glaucoma (NVG); central retinal vein occlusion (CRVO), proliferative diabetic retinopathy (PDR)
Graphical representation comparing the relative 10-year survival rate for all cancer sites combined, the 14 most common cancers, and NVG secondary to PDR and CRVO. NVG secondary to CRVO has a 10-year survival rate of 57.2%. NVG secondary to PDR has a 10-year survival rate of 34.8%, which is the second lowest survival rate with lung and bronchus at 13.8%.
